# Prevalence and Molecular Aspects of Human Hookworms in Guilan Province, Northern Iran

**Published:** 2017

**Authors:** Meysam SHARIFDINI, Laleh GHANBARZADEH, Nasrolla KOUHESTANI-MAKLAVANI, Hamed MIRJALALI, Mehrzad SARAEI

**Affiliations:** 1. Dept. of Medical Parasitology and Mycology, School of Medicine, Guilan University of Medical Sciences, Rasht, Iran; 2. Dept. of Parasitology and Mycology, School of Medicine, Qazvin University of Medical Sciences, Qazvin, Iran; 3. Cellular and Molecular Research Center, Qazvin University of Medical Sciences, Qazvin, Iran; 4. Guilan Province Health Center, Guilan University of Medical Sciences, Rasht, Iran; 5. Foodborne and Waterborne Diseases Research Center, Research Institute for Gastroenterology and Liver Diseases, Shahid Beheshti University of Medical Sciences, Tehran, Iran

**Keywords:** *Necator americanus*, Iran, Prevalence, Phylogenetic analysis

## Abstract

**Background:**

Hookworm infection is one of the important Neglected Tropical Diseases (NTD) in the world. It was previously more prevalent in the northern and southern parts of Iran with a prevalence rate higher than 40% in some endemic regions; nevertheless, the infection rate has decreased to less than 1%. This study aimed to determine prevalence and molecular aspects of hookworm infections in rural inhabitants of Fouman County, Guilan Province, northern Iran

**Methods:**

This cross-sectional study was performed in 31 villages of Fouman district in Guilan Province, northern Iran during 2015–2016. Stool samples were collected from 1500 rural inhabitants and examined by formalin ethyl-acetate concentration as well as agar plate culture techniques. After treatment with albendazole, adult hookworms were isolated. Following DNA extraction, PCR amplification of ITS2-rDNA region was performed and the product was sequenced, followed by genetic variation analysis.

**Results:**

Of 1500 samples, one case was morphologically diagnosed as *N. americanus*. In addition, molecular characterization verified the presence of *N. americanus,* showing more than 95% similarity with sequences of *N. americanus* present in GenBank. The patient showed no clinical symptoms and a mild hypereosinophilia was the only laboratory finding observed.

**Conclusion:**

A reduced prevalence of human hookworms was demonstrated within Guilan Province located in north of Iran. The *N. americanus* originated from Guilan had a high homology with the isolates found in Japan, Laos, Malaysia, and Australia.

## Introduction

Hookworm infection is one of the important Neglected Tropical Diseases (NTD), mainly caused by the nematode parasites *Ancylostoma duodenale* and *Necator americanus* (1). It is distributed in tropical and subtropical countries with high humidity but almost eradicated from Europe and the USA (2, 3). “*N. americanus* is the predominant human hookworm worldwide, whereas *A. duodenale* is found in more scattered focal environments” (4). This infection may cause iron deficiency anemia and other anemia associated-symptoms due to intestinal blood loss and abdominal or epigastric pain, vomiting, loss of appetite, diarrhea and weight loss (4).

*N. americanus* was more prevalent in the Caspian Littoral areas in north of Iran and in the same way *A. duodenale* in the south regions of Iran, previously (5–7). The prevalence of hookworm infection similar to other soil-transmitted helminthes (STHs) has sharply dropped during last decades (8). Lasted studies have shown the prevalence rate of these infections decreased to less than 1% in north and south parts of Iran (9–14).

In recent years, molecular methods have been populated for genetic characterization and phylogenetic analysis of nematodes (15, 16). The internal transcribed spacer 2 (ITS2) region of ribosomal DNA is a useful tool to analyze the genetic variations and phylogenetic relationships in hookworms (17–20).

This work is the first study regarding molecular and phylogenetic analysis of *N. americanus* from Iran. In the current study, the molecular characterization of *N. americanus* isolated from a patient in Fouman district of Guilan province was evaluated based on ITS2 region of ribosomal DNA.

## Materials and Methods

### Study area

**Fig. 1:**
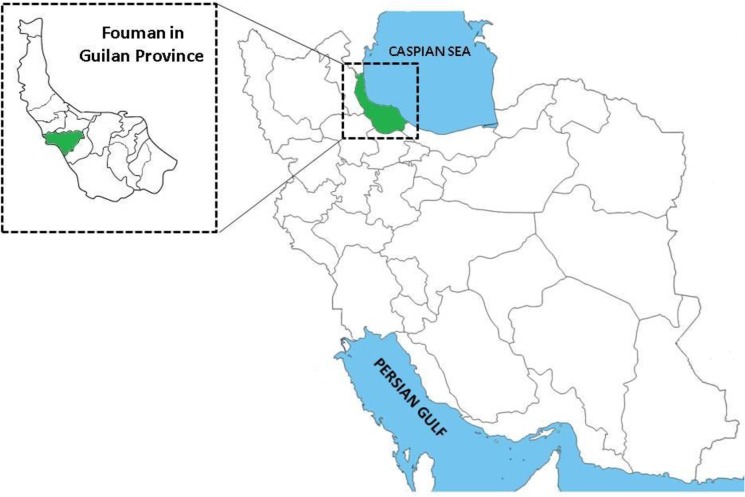
Map of Iran showing geographical location of Guilan Province and the study area, Fouman district

Fouman district in southwestern part of Guilan Province is situated between 37°13′N latitudes and 49°8′E longitudes on the southern part of the Caspian Sea in north of Iran (Fig. 1). It has a humid subtropical climate with annual mean rainfall of 1275 mm. This region of the province is geographically divided into two parts: moderate climate plain and mountainous forest regions (21, 22).

The Ethics Committee of Qazvin University of Medical Sciences, Iran (Ref. No. approved this study protocol IR.QUMS.REC.1394.111).

### Sample collection and parasitological methods

This cross-sectional study was performed in 31 villages of Fouman district in Guilan Province during 2015–2016. Stool samples were randomly collected from 1500 participants (653 males, 547 females) aged 2–87 yr old. All stool samples were examined by formalin ethyl-acetate concentration test. The samples were also subjected to agar plate culture technique as described previously (23). One case of hookworm infection was detected. Next, the patient was treated with 400 mg/day of albendazole for 3 d and stool samples were collected each day, separately. Later, the stool samples were washed through a 50-mesh sieve and observed for detecting adult hookworms using a stereomicroscope.

### Molecular methods

Filariform larvae were collected by washing the surface of agar plate with lukewarm phosphate buffer saline solution and preserved in 70% ethanol (23). Later genomic DNA of the larvae was extracted using DNA Extraction Mini kit (GeneAll, Korea) according to the manufacturer’s protocol and stored at −20 °C until the performance of PCR amplification. Forward primer NC1: 5-ACGTCTGGTTCAGGGTTGTT-3 and reverse primer NC2: 5-TTAGTTTCTTTTCCTCCGCT-3 were used to amplify a 417 bp fragment of ITS2 region of ribosomal gene (24). The PCR amplification was carried out in a final reaction volume of 30 μl containing, 15 μl of PCR mix containing 1.25 U Taq DNA polymerase, 200 μM of dNTPs and 1.5 mM MgCl2 (2× Master Mix RED Ampliqon, Denmark); 10 pmol of each primer and 3 μl of DNA sample. The PCR program was an initial denaturation step at 95 °C for 6 min followed by 35 cycles of 94 °C for 45 sec (denaturation), 60 °C for 90 sec (annealing) and 72 °C for 60 sec (extension), with a final extension at 72 °C for 5 minutes. PCR product was run on a 1.5% agarose gel and visualized using a UV transilluminator (UVItec, EEC). Later, the PCR product was purified using the AccuPrep purification kit (Bioneer, Korea) according to the manufacturer’s instructions and was sequenced in both directions, using forward and reverse primers. The results of sequencing were edited and analyzed by the BioEdit software version 7.2 and the consensus sequences were compared with GenBank reference sequences using the BLAST program (http://www.ncbi.nlm.nih.gov/).

### Phylogenetic analysis

The sequence results were edited and evaluated using the Geneious software (www.geneious.com) and compared with sequences deposited in GenBank database by BLAST program (http://www.ncbi.nlm.nih.gov/). A phylogenetic tree was constructed using the maximum likelihood method based on the Tamura 3-parameter model and pairwise comparisons determined the level of sequence differences within and among species using MEGA 5.0 software (25). Bootstrap analysis was carried out to determine the robustness of the finding based on 1000 replications.

## Results

**Fig. 2:**
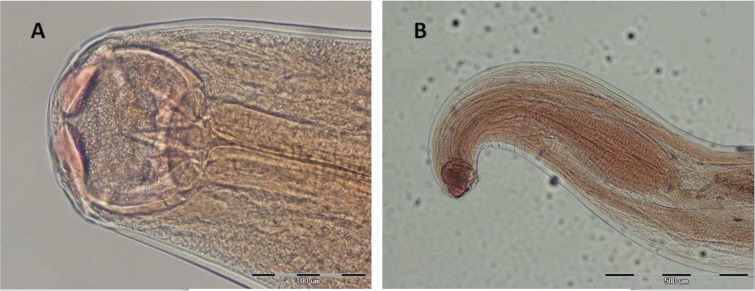
(A, B) Anterior end of *N. americanus* showing cutting plates in mouth capsule with 400× and 100× magnification in A and B, respectively (Original)

**Fig. 3:**
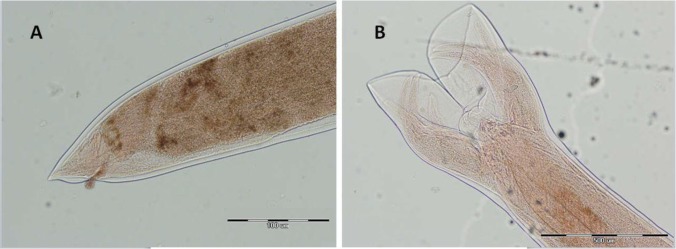
(A) Tail part of female *N. americanus*, (B) caudal bursa of male *N. americanus* with 400× and 100× magnification in A and B, respectively (Original)

Hookworm eggs were detected only in one specimen out of 1500 (0.06%) stool samples collected from villagers. This patient was a 32 yr old male with no significant clinical symptoms. Laboratory data failed to reveal iron-deficiency anemia. The white blood cell count was 5.300/mm^3^ with 54% neutrophils, 43% lymphocytes, 11% eosinophils, and 1% monocytes. Therefore, our patient had only a slight hypereosinophilia. The patient worked as a building constructor and sometimes worked in muddy rice land with barefoot. Adult *N. americanus* including 10 female and 8 male worms were recovered from the patient. Fig. 2 and 3 show the morphological characteristics of male and female worms. In addition, surprisingly, the patient was also infected with *Enterobius vermicularis* and *Giardia lamblia*.

**Fig. 4:**
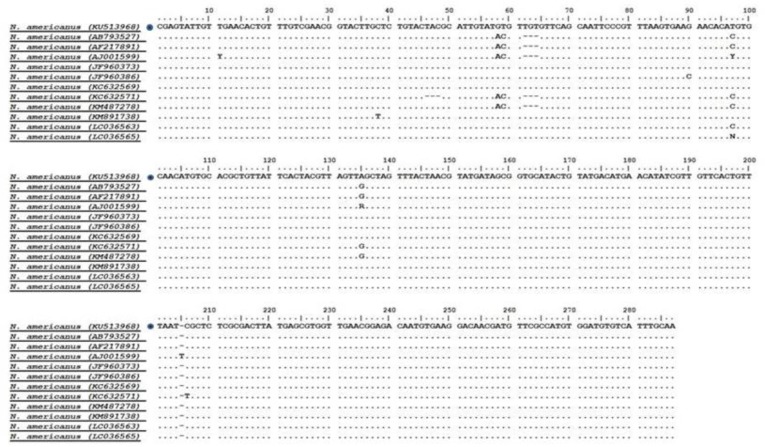
Alignments of the ITS2 sequences for *N. americanus* isolates in the present study (□) and reference sequences retrieved from Gen Bank

The isolate obtained in the current study successfully presented the amplification of about 417 bp for the ITS2 gene. The sequence was compared with other available sequences in the GenBank, using the BLAST system. Accordingly, this isolate demonstrated a high similarity (more than 95%) with *N. americanus* in the GenBank reference sequences. This sequence was deposited in the GenBank database (Accession numbers: KU513968). Fig. 4 shows the sequencing result of *N. americanus* obtained in this study based on the alignment with sequences submitted to the GenBank database.

**Fig. 5:**
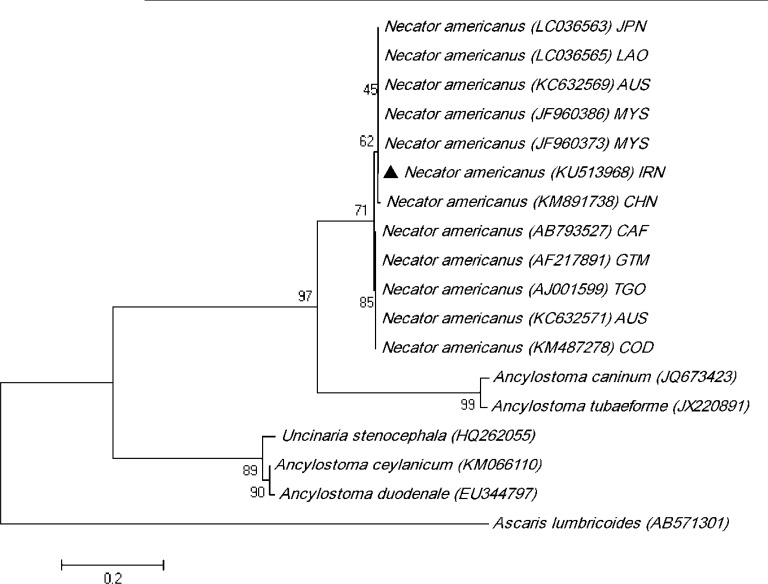
Phylogenetic tree of isolate of *Necator americanus* obtained in this study (▴) and reference sequences retrieved from GenBank based on partial ITS2 sequences and constructed using the Tamura 3-parameter model in MEGA software version 5. GenBank sequences of *N. americanus* included with *Ascaris lumbricoides* as out group. Iran (IRN), Laos (LAO), Malaysia (MYS), Australia (AUS), Japan (JPN), China (CHN), Central African (CAF), Guatemala (GTM), Togo (TGO) and Democratic Republic of the Congo (COD) are represented with country codes (ISO 3166-1 a-3 codes)

Intra-species variation within the isolates of *N. americanus* amounted to 0%–1.7%; meanwhile, inter-species sequence differences among hookworm nematodes were significantly higher, being 1.1%–42.5%. Phylogenetic analysis showed the sequencing result of *N. americanus* obtained in the present study was in the same cluster with sequences from other countries, deposited in the GenBank (Fig. 5).

## Discussion

Hookworms are one of most important STHs with the highest prevalence occurring in sub-Saharan Africa and eastern Asia (1, 26). Hookworm infection in north of Iran was prevalent in the last decades (5, 7). The prevalence of hookworm infection in rural area of Someh-sara district in Guilan province (adjacent region of study area) was 46.6% but none of those patients presented significant anemia because of low intensity of worms (7). In addition, a prevalence rate of 8.9% was reported for the parasite among the rural residents of Lahijan located in the eastern area of Guilan Province (27). In recent decades, prevalence rate of hookworms like other STHs has gradually decreased over time due to improvement in hygienic standards (8).

The main risk factors for hookworm infection are living in rural parts of tropical and subtropical areas with low social and economic levels, poor hygiene, and walking barefoot in the areas high risk for the transmission of hookworms filariform larvae (28, 29). In Guilan Province, the rice lands and tea plantations are the main sources of human hookworm infections because most people are farmers who work in rice fields and tea plantations (7).

The present study showed a limited local transmission of hookworm infection was present in north of Iran during 2015–2016. This transmission of *N. americanus* may be due to the residual of the past high endemicity of hookworm in this area. However, it is a surprising finding for the recent epidemiology of STHs in Iran because hookworm infection was not reported in the endemic areas within recent years (9, 10, 13, 14, 30). Several main factors had the greatest effect on reducing the human hookworms infections in north of Iran, including: 1) protection of water streams from contamination by household excreta; 2) Pesticide spraying of rice fields; 3) Avoiding barefoot walking on soil and muddy areas; 4) promoting health awareness among rural residents concerning the transmission of soil transmitted helminthes, and 5) improving the primary health care (PHC) in rural areas through health houses. In the past, some rural houses, which were adjacent to water streams and their household excreta, discharged into the streams. Thereby, intestinal parasites eggs such as hookworms could be transferred to the rice lands. This inappropriate action of defecation increased the risk of infection by the developed filariform larvae of hookworms in farmers who worked in these areas. Years ago, spraying against pests of rice was not done in the paddy fields in north of Iran, because it was not a main problem for rice production in these areas. After widespread dissemination of rice pests in Guilan and Mazandran Provinces, the chemical control of rice pests by using pesticides have gradually increased years by years. *Chilo suppressalis* that is a stem borer is considered as the most important pest of paddy fields in the north of Iran. Diazinon (O,O-diethyl-O-2-isopropyl-6-methyl (pyrimidine-4-yl) phosphorothioate) and tricyclazole (5-methyl-1,2,4-triazolo (3,4-b)benzothiazole) are organophosphate insecticide and systemic fungicide, respectively, which are used, in addition to other pesticides, for rice in Iran (31). These chemical pesticides have likely lethal effects on developed larvae of hookworms in paddy fields.

The isolate of *N. americanus* obtained from this study had high homology with the isolates of *N. americanus* from Japan (LC036563), Laos (LC036565), Malaysia (JF960373 and JF960386), and Australia (KC632569). We used molecular phylogenetic analysis to determine the evolutionary relationship of the parasite isolated in the present study. The existence of genetic variation among hookworm nematodes has previously been confirmed by the ITS2 region of ribosomal DNA (17–20).

This study is the first phylogenetic analysis of *N. americanus* reported from Iran. The sequences of *N. americanus*, obtained from the current study, were placed in one group along with other *N. americanus* from different countries with high bootstrap support. In this study, phylogenetic analysis indicated that the cluster of Asian isolates of *N. americanus* such as Japan (LC036563), Laos (LC036565) and Malaysia (JF960373 and JF960386) is separated from the rest of the world, including Central African (AB793527), Guatemala (AF217891), Togo (AJ001599) and Democratic Republic of the Congo (KM487278); while the isolates from Australia (KC632569 and KC632571) are located in both branches.

These results show the existence of variation in the ITS2 region sequence in hookworm species, thus the phylogeny analysis of this gene could be a helpful tool in determining the taxonomic classification and geographical location of hookworms.

## Conclusion

The prevalence of hookworms in Guilan Province in north of Iran is extraordinarily reduced and will probably be eliminated within near future. On the other hand, the molecular and phylogenetic tools with ITS2 region showed that the Iranian isolate of *N. americanus* is similar to other Asian isolates.
